# Editorial: Immunity, atherosclerosis, and cardiovascular disease: an interdisciplinary approach to cardiometabolic health

**DOI:** 10.3389/fcvm.2026.1787241

**Published:** 2026-03-09

**Authors:** Sarvesh Chelvanambi, Jürgen Bernhagen, Gabrielle Fredman, Carlos Alberto Labarrere, Holger Winkels, Masanori Aikawa

**Affiliations:** 1 Center for Interdisciplinary Cardiovascular Sciences, Division of Cardiovascular Medicine, Department of Medicine, Brigham and Women’s Hospital, Harvard Medical School, Boston, MA, United States; 2 Division of Vascular Biology, Institute for Stroke and Dementia Research (ISD), LMU Klinikum, Ludwig Maximilian University (LMU) Munich, Munich, Germany; 3 German Center for Cardiovascular Research, Partner Site Munich Heart Alliance, Munich, Germany; 4 Department of Molecular and Cellular Physiology, Albany Medical College, Albany, NY, United States; 5 California Medical Innovations Institute, San Diego, CA, United States; 6 Faculty of Medicine and University Hospital Cologne, Clinic III for Internal Medicine, University of Cologne, Cologne, Germany; 7 Center for Molecular Medicine Cologne, University of Cologne, Cologne, Germany; 8 Center for Excellence in Vascular Biology, Division of Cardiovascular Medicine, Department of Medicine, Brigham and Women’s Hospital, Harvard Medical School, Boston, MA, United States; 9 Channing Division of Network Medicine, Department of Medicine, Brigham Women’s Hospital, Harvard Medical School, Boston, MA, United States

**Keywords:** atheroclerosis, bioinformactics, cardiovascular disease, drug developement, immune system, immunity, interdiscipinarity approach, omics

This Research Topic entitled, “*Immunity, Atherosclerosis and Cardiovascular Disease: An Interdisciplinary Approach to Cardiometabolic Health*,” co-organized by the Frontiers in Cardiovascular Medicine and Frontiers in Immunology published a collection of 19 articles by over 100 authors including both original research reports and review articles. Accumulating evidence suggests that a precision medicine approach to select specific patients suited for optimal therapeutic strategies will empower the next advances in treating cardiovascular disease (1). Many studies have established the role of inflammation in cardiovascular disease (2–4). Understanding how immune cell heterogeneity contributes to cardiovascular diseases, applying diverse approaches is critical to help guide and inform the next generation of immunoregulating therapies (5). This Research Topic covers three general themes. The first theme characterizes immune phenotypes and their contributions to different cardiovascular diseases. The second theme leverages bioinformatic analyses to find novel regulators in cardiovascular diseases which could serve as either diagnostic or therapeutic targets. The third theme focuses on clinical therapeutic strategies aimed at regulating the immune system to reduce the risk of cardiovascular disease progression. Of note, this collection features multiple sub-phenotypes such as distinct pre-existing conditions or co-morbidities associated with atherosclerotic cardiovascular diseases and includes articles addressing the contribution of both the adaptive and innate immune responses in cardiovascular disease. This is of particular importance since immune dysregulation and cardiovascular diseases are chronic processes which require novel and specific opportunities for therapeutic intervention.

## Theme 1: immune phenotypes in cardiovascular diseases

This Research Topic includes eight original reports or review articles that describe immune cell heterogeneity in cardiovascular disease. These articles address cellular phenotypes and signaling axis which influence the immune system during the progression of cardiovascular disease.

Two original research articles reported the contribution of immune cells in cardiovascular diseases. Nartowicz et al. suggested that adults with cyanotic congenital heart disease exhibit a distinct immune phenotype characterized by a notable absence of active inflammatory factors and preserved endothelial function. This profile is biochemically defined by significantly elevated serum levels of sphingosine−1-phosphate, which appears to drive a predominant anti-atherosclerotic activity despite reduced HDL and ApoM concentrations. Liu et al. reported that a low lymphocyte-to-C-reactive protein ratio reflects a pro-inflammatory immune phenotype and independently predicts microvascular obstruction after intervention in ST-segment elevation myocardial infarction (MI). This inflammatory profile links reduced lymphocyte presence with impaired microvascular recovery and improvements in prognostic prediction.

Two review articles addressed distinct immune cell–mediated mechanisms in specific cardiovascular diseases. Yang et al. focused on the crosstalk between cardiac lymphatics and immune cells in the context of MI, proposing potential therapeutic opportunities for regulating these interactions in the heart. Xuan et al. discussed the regulatory roles of various immune cells and the connection between cell death pathways in the microenvironment of the heart following ischemia reperfusion, underscoring immunotherapy as a promising translational approach for MI.

The next set of reviews focused on cells from the innate immune system, specifically macrophages and neutrophils. Ijaz et al. described the heterogeneity within foamy macrophages and their contribution towards progression of atherosclerotic cardiovascular disease. The authors highlighted how these cells exert a diverse set of both anti-inflammatory and pro-inflammatory roles which can be harnessed to develop new therapeutic targets. Jha et al. provided a stellar overview of the concept of elevated uremic toxins in patients with chronic kidney disease and its contribution towards inflammation. Tracking the levels of these uremic toxins and understanding how they impair cardiovascular function could be pivotal for treating complex cardiovascular disease. They specifically focused on how indoxyl sulfate induces pro-inflammatory activation in macrophages to increase the risk of cardiovascular disease. Wang et al. discussed the diverse roles of TREM2 receptor in macrophage signaling in cardiovascular disease, particularly highlighting the protective role of TREM2 signaling as an interesting candidate for therapeutic intervention. Lian et al. addressed key role of neutrophils and the innate immune system in the development and progression of cardiovascular diseases. They described the diverse phenotypes of neutrophils and signaling factors that promote neutrophil recruitment and activation, suggesting that regulating neutrophil phenotypes could help to prevent progression in cardiovascular diseases.

## Theme 2: omics and bioinformatics in cardiovascular diseases

The second theme in this Research Topic featured the analysis of omics data supported by machine learning and bioinformatics to identify novel contributors to cardiovascular disease progression related to immune dysregulation. Four original research studies used a similar philosophy to leverage publicly available datasets to re-analyze and identify different disease specific contributors. Zhang et al. integrated machine learning with bioinformatic analysis to identify three gene signatures in atherosclerosis with ischemic stroke. The authors proposed that OAS2, TMEM106A and ABCB1 could serve both as diagnostic markers and as tools to track immune cell involvement in atherosclerosis. Jin et al. used bioinformatics and machine learning to identify diagnostic biomarkers and immune cell infiltration in unstable coronary atherosclerotic plaques to help improve classification of patient subgroups. Qing et al. compared datasets from atherosclerosis and gout to identify common genes. Machine learning-based bioinformatic analysis enabled to select genes which are strongly linked with a contribution of aggravated inflammation in both conditions. Using machine learning and bioinformatics, Jiang et al. identified N6-methyladenosine related ferroptosis associated genes in the context of atherosclerosis.

One review article by Thorp et al. complements this theme and discusses how multi-dimensional omics data helps to shed new light to determine the contribution of immune cells to cardiovascular disease progression. The authors provided an in-depth review of chronic allograft vasculopathy in heart transplant recipients and summarized the current state of multi-omic and single-cell studies in this context, highlighting that these advanced approaches have shed light into the role played by immune cell subpopulations in disease progression.

## Theme 3: therapeutic approaches to target immune systems for cardiovascular diseases

The third theme features five review articles describing potential therapeutic strategies to target immune regulation and reduce the risk for cardiovascular disease progression.

The systematic review by Chen et al. evaluated 20 meta-analyses on the use of traditional Chinese patented oral medicines that promote blood circulation in parallel with Western medicine for the treatment of angina pectoris. The authors noted that the methodological quality of many of these studies is poor, complicating accurate comparison and integration difficult and highlighted potential templates for future studies to help facilitate such comparisons. In another systematic review by Bekbossynova et al., the authors investigated the common genetic variants in patients with familial hypercholesterolemia and their contribution to carotid atherosclerosis progression. Based on 9 different trials, the authors concluded that LDL-receptor mutations influence the progression of carotid artery atherosclerosis. Wang et al. examined whether subclinical hypothyroidism might increase the risk of cardiovascular disease. Although the authors observed associations between dyslipidemia and carotid intima–media thickness, they concluded that additional studies are required to establish a causal relationship. Giakomidi et al. explored how the gut microbiome release metabolites that regulate the adaptive immune system. In addition, the effect of these metabolites to impact both innate and adaptive immune system could increase the rate of progression of atherosclerotic disease. The authors suggested that the use of such information helps to modulate the immune system in atherosclerosis. Skrobucha et al. explored the use of GLP-1 as a peptide hormone to reduce the incidence of coronary artery disease. The authors demonstrated evidence from multiple animal studies for GLP-1-driven anti-inflammatory effects for reduction of cardiovascular disease risk. Human studies are limited in size and scope to determine these effects while some evidence on the benefits for endothelial function has been reported. The authors suggested that future studies in larger randomized controlled trials with sufficient outcomes measured could help inform of the benefits of GLP-1 treatment to reduce cardiovascular disease burden. The meta-analysis by Xie et al. evaluated 42 randomized controlled trials to determine the effect of sodium tanshinone IIA sulfonate injection to treat cardiovascular disease. The authors suggested that this ingredient found in traditional Chinese medicine across 42 different studies reduced pro-inflammatory markers in patients with atherosclerosis but its specific contribution to reducing cardiovascular disease risk burden is yet to be fully elucidated.

## Concluding remarks

This Research Topic brought together 19 articles that define immune phenotypes, leverage omics-powered discovery research, and propose new immunomodulatory therapeutics for cardiovascular diseases. Collectively, these articles highlight the central role of immune cell heterogeneity, regulatory mechanisms at the systems level, and translational potential to slow disease progression. Integrating multi-omic profiling with precise patient stratification will be critical for translating immune-targeted interventions into effective precision therapies for inflammatory cardiovascular disease (6).

## References

[1] Aikawa M Sonawane AR Chelvanambi S Asano T Halu A Matamalas JT Precision cardiovascular medicine: shifting the innovation paradigm. Front Sci. (2025) 3:1474469. 10.3389/fsci.2025.1474469 41378166 PMC12687475

[2] Schelemei P Wagner E Picard FSR Winkels H. Macrophage mediators and mechanisms in cardiovascular disease. FASEB J. (2024) 38(2):e23424. 10.1096/fj.202302001R 38275140

[3] Libby P Soehnlein O. Inflammation in atherosclerosis: lessons and therapeutic implications. Immunity. (2025) 58(10):2383–401. 10.1016/j.immuni.2025.09.012 41045921

[4] Fredman G Serhan CN. Specialized pro-resolving mediators in vascular inflammation and atherosclerotic cardiovascular disease. Nat Rev Cardiol. (2024) 21(11):808–23. 10.1038/s41569-023-00984-x 38216693 PMC12863063

[5] Chelvanambi S Decano JL Winkels H Giannarelli C Aikawa M. Decoding macrophage heterogeneity to unravel vascular inflammation as a path to precision medicine. Arterioscler Thromb Vasc Biol. (2024) 44(11):2253–7. 10.1161/ATVBAHA.124.319571 39441912 PMC11715277

[6] Giugni FR Berry JD Khera A Shah AM de Lemos JA. Precision medicine for cardiovascular prevention and population health: a bridge too far? Circulation. (2024) 150(21):1720–31. 10.1161/CIRCULATIONAHA.124.070081 39556656 PMC11575940

